# Chlorogenic Acid Alleviates Colon Mucosal Damage Induced by a High-Fat Diet via Gut Microflora Adjustment to Increase Short-Chain Fatty Acid Accumulation in Rats

**DOI:** 10.1155/2021/3456542

**Published:** 2021-02-01

**Authors:** M. Gui Xie, Y. Quan Fei, Y. Wang, W. Yan Wang, Z. Wang

**Affiliations:** ^1^College of Bioscience and Biotechnology, Hunan Agricultural University, Changsha, Hunan 410128, China; ^2^College of Agriculture and Biotechnology, Hunan University of Humanities, Science and Technology, Loudi 417000, China

## Abstract

A high-fat diet (HFD) has been previously associated with the development of diseases such as chronic colitis. While chlorogenic acid (CGA) is known to exhibit potent antioxidant, antibacterial, and anti-inflammatory properties, little is known about its effects on intestinal inflammation. In this study, we investigated the effects of CGA on intestinal inflammation in an HFD-induced obesity rat model and assessed whether these effects were related to changes in gut microbiota composition. This was achieved by examining physiological and biochemical indicators, the liver transcriptome, and the structure of the fecal microflora. CGA treatment significantly reduced HFD-induced internal organ weight gain, promoted colon tissue repair, downregulated the expression of inflammatory cytokines, and promoted the accumulation of the tight junction protein. KEGG enrichment analysis of differentially expressed genes, applied to data from the RNA-seq of rat liver tissue, revealed that CGA treatment significantly affected amino acid and lipid metabolism in the liver. Furthermore, CGA decreased the abundance of bacteria belonging to the genera *Blautia*, *Sutterella*, and *Akkermansia* and increased butyric acid levels, which were positively correlated with *the abundance of Ruminococcus* (butyric acid producer). Moreover, the beneficial changes observed in the HFD group were not as pronounced as those in the CGA treatment group. In summary, CGA can alleviate colitis in HFD-induced obesity through its anti-inflammatory effects associated with changes in gut microbiota composition and an increase in the production of short-chain fatty acids and thus can be used as a potential drug for the treatment of this pathology.

## 1. Introduction

Obesity has become a global epidemic, increasing in prevalence in both developed and developing countries. Moreover, the current trend shows that obesity develops at an earlier age in humans [1]. Approximately 937 million obese and 396 million overweight adults were reported globally in 2005, and these numbers are predicted to increase to 1.35 billion and 573 million, respectively, by 2030 [2]. A long-term high-fat diet (HFD) can lead to obesity [1], metabolic disorders, and an increased risk of developing a series of chronic diseases, such as type II diabetes or cardiovascular disease [3]. Structural and functional changes in the intestinal microbiota occur as a result of HFD intake, leading to an increase in the accumulation of inflammation-promoting metabolites, which in turn contribute to an increased risk of developing colorectal cancer [4].

Several studies have shown that food polyphenols influence the community structure and morphology of the intestinal microflora [5, 6]. For example, resveratrol regulates intestinal microflora composition as well as lipogenesis by suppressing lipoprotein lipase expression and the expression of genes involved in fatty acid biosynthesis [5]. Tea polyphenols improve dysbiotic intestinal flora by upregulating antioxidant enzyme activity and tight junction protein levels in the ileum [7].

Chlorogenic acid (CGA) is a well-known phenolic acid derived from caffeic acid and quinic acid [8] and is widely found in plants, fruits, and vegetables [9]. It has antibacterial, antiviral, antitumor, and antioxidant properties. Furthermore, it has been reported to lower blood pressure and blood lipid levels, increase white blood cell counts, protect the liver and gallbladder, and scavenge free radicals [10, 11]. Our previous studies have shown that CGA supplementation in HFD-fed rats can decrease body weight and improve lipid metabolism disorders and obesity-related hormone levels. Furthermore, CGA supplementation reduces macrophage infiltration and suppresses inflammatory signaling [12]. Additionally, CGA can attenuate colon barrier damage by decreasing myosin light chain kinase expression and promoting the dynamic distribution of tight junction proteins in a colitis rat model [13]. CGA supplementation also ameliorates 2.5% dextran sulfate sodium-induced colitis by suppressing signaling pathways involved in the inflammatory response and apoptosis [14]. Long-term CGA supplementation increases gut microbiota diversity, thereby potentially improving overall metabolism. Furthermore, chronic dietary CGA attenuates diet-induced inflammation as well as cardiovascular, liver, and metabolic changes [15].

The HFD-induced obesity rat model has been widely used to study insulin resistance, blood glucose homeostasis, dyslipidemia, and genetic fatty liver disease [16–19]. However, few studies have investigated the effects of CGA on intestinal inflammation induced by HFD. Therefore, we aimed to investigate the ability of CGA to slow down or inhibit intestinal inflammation in rats after 7 weeks of HFD feeding. For this purpose, rats were divided into three groups (HFD, HFD+SPR (high-fat recovery group), and HFD+CGA). Furthermore, we compared the effects of natural recovery and CGA treatment on HFD-induced colon inflammation by analyzing physiological and biochemical indices, the liver transcriptome, and the intestinal flora of rats. Finally, we aimed to explain the underlying mechanisms of action of CGA.

## 2. Materials and Methods

### 2.1. Animal Experiments

**Figure 1:**
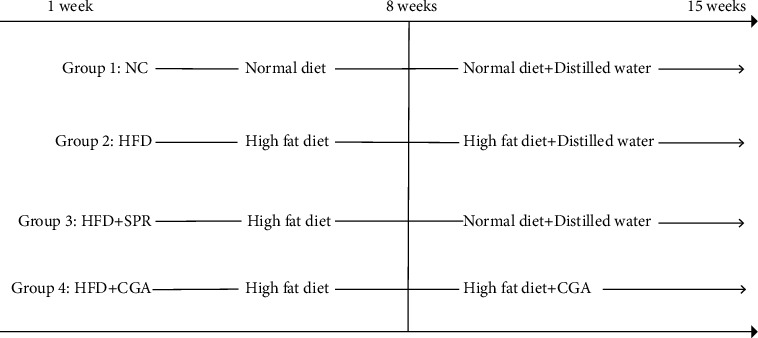
Time chart of the experimental treatment plan.

All animal experiments were in accordance with the Guide for the Care and Use of Laboratory Animals of Hunan Agricultural University and performed in compliance with the Guide for the Protection and Use of Laboratory Animals of Hunan Agricultural University. The protocol was approved by the Animal Protection and Utilization Committee of Hunan Agricultural University. Forty male Sprague-Dawley (SD) rats (Hunan Silaike Jingda Co., Changsha, China; certificate number, HNASLKJ2016-0002) were reared in the animal room of the Institute of Subtropical Agricultural Research, Chinese Academy of Sciences, with two rats housed in each cage. All rats were provided free access to food and water throughout the experiment. The room was maintained at 22 ± 1°C and approximately 60% humidity. After one week of adaptive feeding under a 12-hour light/dark cycle, the rats were randomly divided into the following two groups: normal control group (NC; *n* = 10) and high-fat diet group (HFD; *n* = 30). After 7 weeks, the rat obesity model was established (the average body weight of rats in the HFD group was >20% higher than the average body weight of the NC group [20]). The body weight of rats in the NC group was unaltered. Next, the HFD group was equally divided into the following three subgroups: the HFD group (*n* = 10, fed a high-fat diet, with distilled water administered by gavage), the high-fat recovery group (HFD+SPR; *n* = 10, switched to normal feed, with distilled water administered by gavage), and the CGA group (HFD+CGA; *n* = 10, fed a high-fat diet, with 100 mg/kg CGA (CAS: 327-97-9) administered by gavage). The NC group was fed a diet of 3.6 kcal/g, containing 72.3% carbohydrates, 19.7% protein, and 10% fat. The HFD group received a diet of 4.6 kcal/g, containing 46.4% carbohydrates, 19.6% protein, and 34.6% fat. Chow was ordered from Botai Hongda Biotechnology Co. (Beijing, China).  Figure 1 provides a schematic diagram of the treatment schedule. Gastric perfusion was performed after 8 weeks of experimentation. In the later stage of treatment, rat feces were collected after fasting for 12 h before administering the gavage. The collected fecal samples were placed in a sterile enzyme-free Eppendorf tube and stored at -80°C. Blood samples were collected from the heart, incubated at room temperature for 30 min, and centrifuged at 3,500 rpm at 4°C for 15 min. The isolated serum was stored at -80°C. At the end of the experimental period, the rats were sacrificed using pentobarbital sodium. Then, the rats were dissected, and tissues and organs of interest were removed, weighed, washed with normal saline, and preserved in liquid nitrogen. Parts of the colon and liver tissues were removed and fixed in 10% formalin for subsequent histopathological analysis.

### 2.2. Histomorphological Examination

The colon and liver tissues from each group (*n* = 6) were fixed in 10% formalin. Tissue samples were slowly flushed with water, dehydrated in different concentrations of ethanol at 37-45°C for 2-4 h, and embedded in wax (SVA, Uppsala, Sweden). Tissue was cut and sectioned into 5 *μ*m sample slices, dewaxed, stained, and sealed. Cross-sections of colon and liver tissues were photographed under a 10x and 40x objective lens using a biomicroscope (ML31; MSHOT, Guangzhou, China), and gross pathological changes in the tissue were observed and recorded.

### 2.3. Enzyme-Linked Immunosorbent Assays

For the four groups, plasma levels of tumor necrosis factor-alpha (TNF-*α*), monocyte chemoattractant protein-1 (MCP-1), interleukin 6 (IL-6), and lipopolysaccharide (LPS) were measured using commercially available kits (CSB-E14247r/E04640r/E04595r/E11987r/E07429r; CUSABIO, Wuhan, China) and a microplate reader (BioTek, Winooski, VT, USA).

### 2.4. Quantitative PCR Analysis

Total RNA was extracted from colon tissues using TriQuick Reagent (Solarbio, Beijing, China), and the obtained precipitate was washed and dissolved. RNA quality was assessed using an ultra-micro UV-visible spectrophotometer (NanoDrop 2000; Thermo Fisher Scientific, Waltham, MA, USA). RNA was reverse-transcribed into cDNA, and quantitative PCR was performed. For all samples, the RNA concentration was adjusted to 1,000 ng/*μ*L. Reverse-transcription was performed using a FastKing RT Kit with gDNase (TaKaRa, Kusatsu, Japan) to remove genomic DNA, in accordance with the manufacturer's instructions. A SuperReal PreMixPlus SYBR Green kit (TIANGEN, Beijing, China) was used for quantitative RT-PCR. Ct values were generated for *TNF-α*, *MCP-1*, *IL-6*, and *β-actin* from rat colon tissue using an ABI 7300 Real-Time PCR Instrument (Applied Biosystems, Foster City, CA, USA). Relative expression was determined using the 2^-*ΔΔ*Ct^ method.

### 2.5. Determination of Protein Expression Levels by Western Blotting

After thoroughly grinding the protein samples with a liquid, RIPA buffer solution was added (a piece of protease inhibitor was added per 10 mL of RIPA buffer solution), followed by lysis on ice for approximately 30 min. The lysed sample was centrifuged at 12,000 rpm at 4°C for 5 min, and the supernatant was collected into a new EP tube. The protein concentration was determined using a BCA protein concentration determination kit (Beyotime Biotechnology, Shanghai, China). The concentration of all samples was adjusted to 1000 ng/*μ*L, and samples were transferred into new EP tubes. Next, the samples were denatured in a water bath at 99°C for 10 min, immediately cooled to 4°C in ice (liquid water vapor was removed from the tube wall), shaken and mixed thoroughly, and finally aliquoted and stored at -80°C until western blotting. Based on the molecular weight of the selected antibody, the concentration of the resolving (lower) gel was 12% and that of the stacking (upper) gel was 5%. Electrophoresis was performed at 80 V for approximately 20 min, with the sample separating in the separation gel. The voltage was increased to 120 V, and electrophoresis was continued for approximately 1 h. The proteins were transferred from the gel onto blotting membranes at 180 mA for 75 min and blocked with skimmed milk powder for 2 h. The membrane was washed once with TBST (Beyotime Biotechnology, Shanghai, China), and the corresponding primary antibody (Proteintech, Chicago, USA) was applied overnight (the dilution ratio of TNF-*α* (17590-1-AP), IL-10 (60269-1-Ig), Ocln (13409-1-AP), IL-6 (66146-1-Ig), and *β*-actin (66009-1-Ig) was 1 : 1000). Then, the primary antibody was washed off, and the secondary antibody was applied for 2 h (the dilution ratio of Goat anti-rabbit IgG (H + L) (SA00002-2) and Goat anti-rat IgG (H + L) (SA00002-9) was 1 : 5000). Before chemiluminescent detection, the membrane was washed and the color was developed with a luminous chromogenic solution. Results were analyzed using ImageJ (NIH, Bethesda, MD, USA), converting the destination strip image to grayscale image, compared the intensity of destination strips with *β*-actin strip as the control.

### 2.6. Liver Transcriptome Analysis by RNA-seq

Total RNA was extracted from the liver tissue using TriQuick Reagent (Solarbio, Beijing, China). RNA quality was then assessed using the ultra-micro UV-visible spectrophotometer Agilent 2100 Bioanalyzer (Agilent RNA 6000 Nano Kit), and RNA integrity was determined by gel electrophoresis. Next, quality control analysis of raw reads was performed to determine whether the sequenced data were suitable for the follow-up analysis (https://github.com/BGI-flexlab/SOAPnuke). Hierarchical Indexing for Spliced Alignment of Transcripts was used to align reads with the reference genome in directional mode (http://www.ccb.jhu.edu/software/hisat). Clean reads were mapped to the reference genome using Bowtie 2 (http://bowtie-bio.sourceforge.net/Bowtie2/index.shtml), and then, gene expression levels were calculated with RSEM (http://deweylab.biostat.wisc.edu/ RSEM). We detected DEGs with DEseq2, which is based on the negative binomial distribution (https://bioconductor.org/packages/release/bioc/html/DESeq2.html). Based on KEGG annotation results, we classified DEGs according to the official classification and performed KEGG pathway functional enrichment using phyper, a function of R.

### 2.7. Determination of Short-Chain Fatty Acid Levels in Feces by GC-MS

In brief, rat feces were added to 1 mL of ddH_2_O, mixed thoroughly on a vortex mixer, and shaken on an oscillator for 30 min. Then, the samples were incubated overnight at 4°C, after centrifugation at 15,000 rpm for 20 min, and the supernatant was transferred to a new EP tube. The supernatant was mixed with 25% metaphosphoric acid at a volume ratio of 9 : 1 and then reacted at room temperature for 4 h. The samples were centrifuged at 12,000 rpm for 15 min, and the resulting supernatant was filtered through a 0.45 *μ*m disposable water membrane and added to an N10149 automatic liquid sampler (Agilent, Santa Clara, CA, USA) for evaluation by gas chromatography-mass spectrometry (GC-MS) to create a standard curve for analyzing various known short-chain fatty acids (SCFAs). Each stock solution of different SCFAs (Sigma, St. Louis, MO, United States) was prepared, and gradient of 10 *μ*L, 20 *μ*L, 50 *μ*L, 100 *μ*L, 300 *μ*L, and 500 *μ*L of stock solutions were, respectively, mixed with 100 *μ*L of 25% metaphosphoric acid, then diluted to 1000 *μ*L with ddH_2_O. The stock solution and diluted solution were stored at 4°C away from light. Chromatographic analysis was determined using DB-FFAP column of 30 m (length) × 0.25 *μ*m (inner diameter) × 0.25 *μ*m (film thickness) with a flame ionization detector (FID), and high purity nitrogen (99.999%) was used as the carrier gas at a flow rate of 0.8 mL/min, and high purity hydrogen (99.999%) was used as the auxiliary gas. The initial temperature was 60°C, and the temperature increased to 220°C at a rate of 20°C/min and maintained for 1 min.

### 2.8. Determination of Intestinal Microorganisms

DNA was extracted from rat feces using a DNA Stool Mini Kit (Qiagen, Hilden, Germany). DNA sample integrity was assessed by 1% agarose gel electrophoresis, with a NanoDrop 2000 spectrophotometer used to determine DNA concentration and purity. The V3 and V4 hypervariable regions of 16S rRNA were selected for DNA amplification. The NEB Next Ultra DNA Library Prep Kit for Illumina (New England Biolabs, Ipswich, MA, USA) was used to construct the library, and libraries were sequenced on a MiSeq instrument (Illumina, San Diego, CA, USA) after passing Qubit quantification and the library test specifications. Raw sequencing data were spliced and filtered to obtain clean reads. Paired-end sequencing fragments were spliced using Adobe Flash Professional, with a threshold of 97% similarity. Operational taxonomic unit (OTU) clustering and species analysis were performed. The diversity of the sequence data was analyzed using QIIME software, and sample richness and evenness information were obtained. Additionally, the samples were analyzed by weighted principal coordinate analysis (PCoA) and clustered based on UniFrac distance. The community structure among different samples and groups was analyzed. All offline data were analyzed by the Beijing Genomics Institute.

### 2.9. Statistical Analysis

Statistical analysis was performed using SPSS 25.0 software (IBM, Armonk, NY, USA), and single-factor analysis of variance was used to analyze data from the same group and different groups. Data are expressed as mean ± SEM. Data from multiple groups were analyzed by analysis of variance. The results were analyzed and plotted using Prism 7 (GraphPad, San Diego, CA, USA).

## 3. Results and Discussion

### 3.1. Effects of High-Fat Diet on Body Weight and Body Fat Content of SD Rats

**Figure 2:**
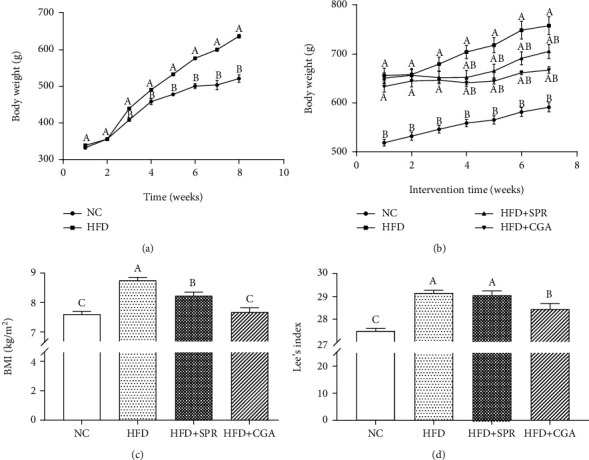
Effect of chlorogenic acid on body index values: (a) weight changes during the establishment of the model; (b) weight changes during chlorogenic acid (CGA) treatment; (c) BMI; (d) Lee's index. NC: natural control group; HFD: high-fat diet group; HFD+SPR: high-fat recovery group; HFD+CGA: CGA treatment group. All data are expressed as mean ± SEM. The values in the same row with different letters are significantly different (^∗^*P* < 0.05), and the values of each group with the same letters are not significantly different (*P* > 0.05).

In terms of the initial body weight, no significant difference was observed between groups (the average initial weight was 338.45 ± 14.8 g). After 2 weeks of feeding the HFD, body weights were significantly different between the NC and HFD groups (*P* < 0.05). As shown in Figure 2(a), after 8 weeks of HFD feeding, the average body weight of rats in the HFD group was more than 20% higher than the average body weight of the NC group, indicating that the obesity model was successfully established. The weight change in rats receiving CGA treatment is shown in Figure 2(b). No significant difference was observed between the four treatment groups; however, compared with the HFD group, the weight of rats in the HFD+SPR and HFD+CGA groups increased at a slower rate. The effects of CGA on BMI and Lee index values are presented in Figures 2(c) and 2(d). CGA treatment significantly reduced the BMI (*P* < 0.05) and Lee index (*P* < 0.05) values of HFD rats. Furthermore, the HFD+SPR group presented significantly reduced BMI values (*P* < 0.05) and Lee index values, to a certain extent. The various physical indicators of SD rats in each group are shown in Table 1. Compared with the NC group, the weight of all important organs, except the testicular tissue in the HFD group, increased significantly; compared with the HFD group, the weight of all important organs, except testicular tissue, decreased significantly after CGA treatment. The increased weight of all internal organs demonstrated that the obesity model was successful. Aspartate aminotransferase (AST) and alanine aminotransferase (ALT) values assess the extent of liver damage, whereas immunoglobulin G (IgG) and IgM determine the presence of inflammation as their expression is elevated during inflammatory conditions. Typically, low-density lipoprotein cholesterol (LDL-C) and high-density lipoprotein cholesterol (HDL-C) are used to assess high-fat obesity models and, hence, were accordingly selected to model obesity. Additionally, cholesterol values are assessed to examine cholesterol metabolism in rats. Compared with the NC group, the plasma levels of LDL-C, HDL-C, IgG, IgM, AST, and ALT decreased significantly in the HFD group. Moreover, the declining LDL-C/HDL-C ratio, often considered to attenuate the risk of metabolic disorders associated with obesity [21], indicated that the HFD disrupted the blood lipid metabolism in rats to a certain extent; however, following CGA treatment, the above indices were significantly improved. These results showed that CGA can alleviate obesity caused by the HFD, demonstrating efficacy consistent with the effects of dietary correction (HFD+SPR).

### 3.2. Histopathological Analysis Showed That Chlorogenic Acid Reduces Colonic and Liver Tissue Inflammation Caused by a High-Fat Diet

**Figure 3:**
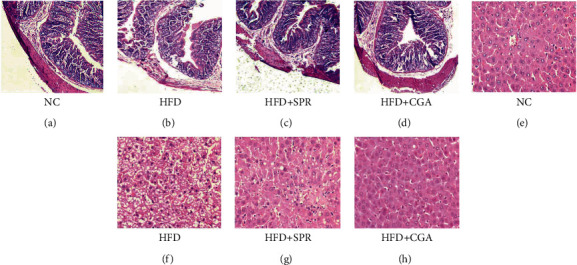
Hematoxylin-eosin staining of rat colonic and liver tissue from each group. Hematoxylin-eosin staining of colonic and liver tissue of rats from the natural control (NC, (a, e)), high-fat diet (HFD, (b, f)), high-fat recovery (HFD+SPR, (c, g)), and chlorogenic acid treatment (HFD+CGA, (d, h)) groups. Stained colonic and liver tissue sections were photographed using a Mingmei ML31 biomicroscope under 10x and 40x objective lens.

Figure 3 presents the hematoxylin and eosin (HE) staining of rat colon and liver tissue sections of each group. In the NC group (Figure 3(a)), the colon epithelium was intact and the crypt structure appeared normal. In the HFD group (Figure 3(b)), the colon epithelial mucosa was slightly swollen, with damaged and irregular crypts. In the HFD+SPR group (Figure 3(c)), the colon crypts presented a relatively compact structure, without significant damage, which was significantly improved when compared with the HFD group. In contrast, in the HFD+CGA group (Figure 3(d)), the colon epithelial mucosa was slightly ulcerated, the colon epithelial tissue was damaged, the crypt structure was normal, and no inflammation-associated infiltrations were observed. Figures 3(e)–3(h) present the HE-stained micrographs of rat liver tissues. A normal phenotype was observed in the liver tissues of normal rats (Figure 3(e)). However, liver tissue sections from HFD-fed rats (Figure 3(f)) showed enlarged hepatocytes, disorganized vacuolar degeneration, and deposits of fatty granules. The HFD-specific vacuolar phenotypes were significantly alleviated in the HFD+SPR group (Figure 3(g)), while the hepatocyte size was normal in the HFD+CGA group (Figure 3(h)). Therefore, CGA treatment can significantly reduce HFD-induced colon inflammation in SD rats.

### 3.3. Effect of Chlorogenic Acid on Serum LPS Levels and the Expression of Related Inflammatory Cytokines

**Figure 4:**
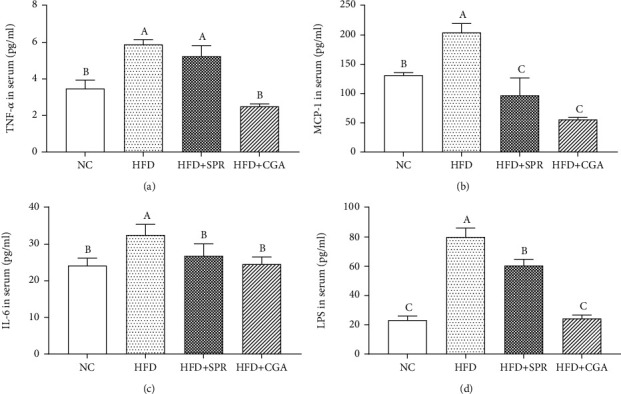
Effects of chlorogenic acid on the expression of inflammatory cytokines and LPS. Serum levels of TNF-*α* (a), MCP-1 (b), IL-6 (c), and LPS (d). NC: natural control group; HFD: high-fat diet group; HFD+SPR: high-fat recovery group; HFD+CGA: chlorogenic acid treatment group.

Serum inflammatory cytokine levels for each animal group are shown in Figure 4. Compared with the NC group, the levels of the proinflammatory factors, TNF-*α*, MCP-1, IL-6, and LPS, increased by 69.63%, 94.99%, 59.39%, and 207.33%, respectively, in the HFD group. The levels of TNF-*α*, MCP-1, IL-6, and LPS in the HFD+SPR and HFD+CGA groups were significantly lower than those observed in the HFD group (*P* < 0.05); in the HFD+SPR group, the levels of these factors decreased by 12.37%, 225.95%, 53.52%, and 302.93%, respectively, and in the HFD+CGA group, the levels were decreased by 135.23%, 308.16%, 66.78%, and 265.34%, respectively. These results revealed that CGA can relieve inflammation caused by an HFD, and its effect was superior to that demonstrated by dietary correction.

### 3.4. Effect of Chlorogenic Acid on Cytokine Gene Expression in Colonic Tissue

**Figure 5:**
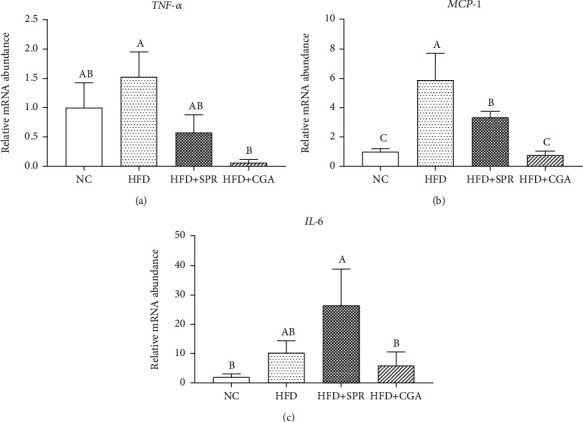
Expression levels of related genes in colonic tissue of rats in each group. Relative mRNA expression of *TNF-α* (a), *MCP-1* (b), and *IL-6* (c) in colonic tissue of rats.

The relative mRNA expression levels of related genes in rat colonic tissues for each group are shown in Figure 5. The relative mRNA expression levels of *TNF-α* and *MCP-1* were significantly higher in the HFD group than in the NC group, with lower expression levels observed in the HFD+CGA group than in the HFD+SPR group. Compared with the NC group, the relative mRNA expression levels of *TNF-α* increased by 52.64% in the HFD group, and the relative mRNA expression levels of *MCP-1* increased by 488.48%. Compared with the HFD group, the relative mRNA expression levels of *TNF-α*, *MCP-1*, and *IL-6* decreased by 2365.92%, 673.30%, and 73.58%, respectively, in the HFD+CGA group, which significantly differed from expression levels in the HFD group. In summary, CGA treatment inhibited colonic tissue inflammation in rats fed an HFD.

### 3.5. Effect of Chlorogenic Acid on Cytokine Protein Expression in Colonic Tissue

**Figure 6:**
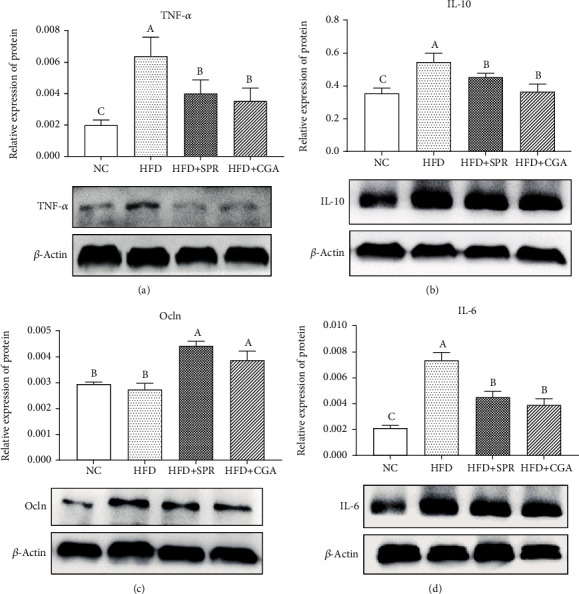
Protein expression levels in colonic tissue of rats in each group. Protein expression levels of TNF-*α* (a), IL-6 (b), Ocln (c), and IL-10 (d) in rat colonic tissue.

The expression levels of relevant proteins in rat colonic tissues from each group are shown in Figure 6. No significant differences in TNF-*α* protein expression levels were observed among the NC, HFD+SPR, and HFD+CGA groups; however, TNF-*α* protein expression levels were significantly lower in the HFD+CGA and HFD+SPR groups than in the HFD group. The protein expression of IL-6 was significantly higher in the HFD group than in the NC group and the HFD+CGA group (*P* < 0.05). CGA may have inhibited the expression of IL-6 protein and alleviated colonic inflammation to a certain extent. Compared with the NC group, the expression of Ocln, an intestinal epithelial tight junction protein, was significantly higher in the HFD+SPR and HFD+CGA groups (*P* < 0.05). IL-10 expression levels were higher in the HFD+CGA group than in the NC group; however, inflammation induced by hyperlipidemia was not significantly alleviated following CGA treatment.

### 3.6. KEGG Pathway Analysis of Differentially Expressed Genes in Liver Tissue

**Figure 7:**
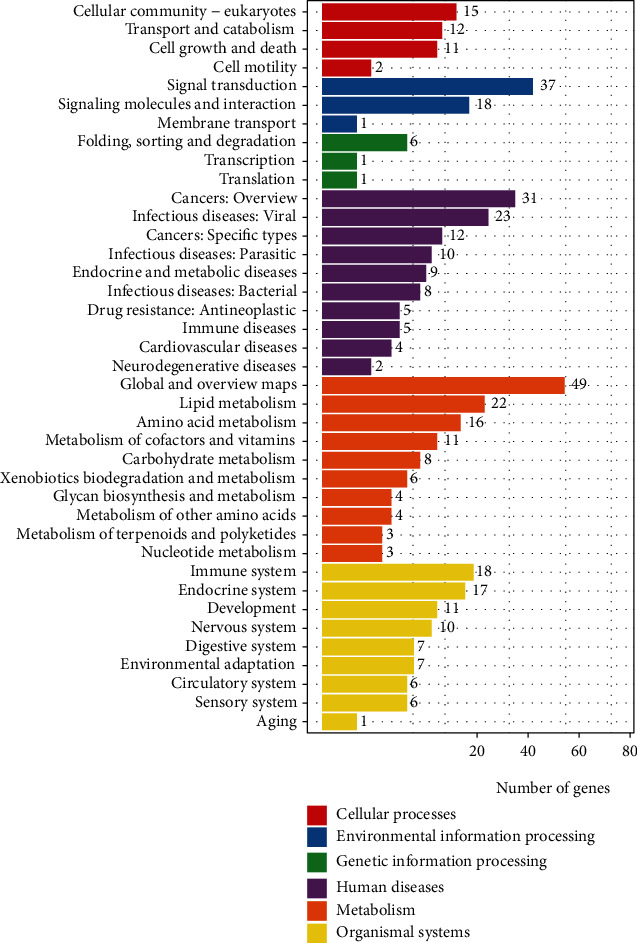
The classification of biological pathways of differential genes between the HFD and HFD+CGA groups.

Based on the classification and enrichment analysis of KEGG biological pathways, the classification of biological pathways of differential genes between the HFD and HFD+CGA groups was enriched (Figure 7). The pathways were divided into six categories including human diseases, metabolism, organismal systems, cellular processes, and environmental and genetic information processing. A total of 422 differentially expressed genes were involved in 208 pathways between the HFD group and the HFD+CGA group, of which 41 pathways were significantly enriched, including 11 significant signaling pathways related to amino acid and lipid metabolism, listed top place (Table 2). Additionally, the bile acid excretion pathway was also included.

### 3.7. Effect of Chlorogenic Acid on Intestinal Microbial Diversity

**Figure 8:**
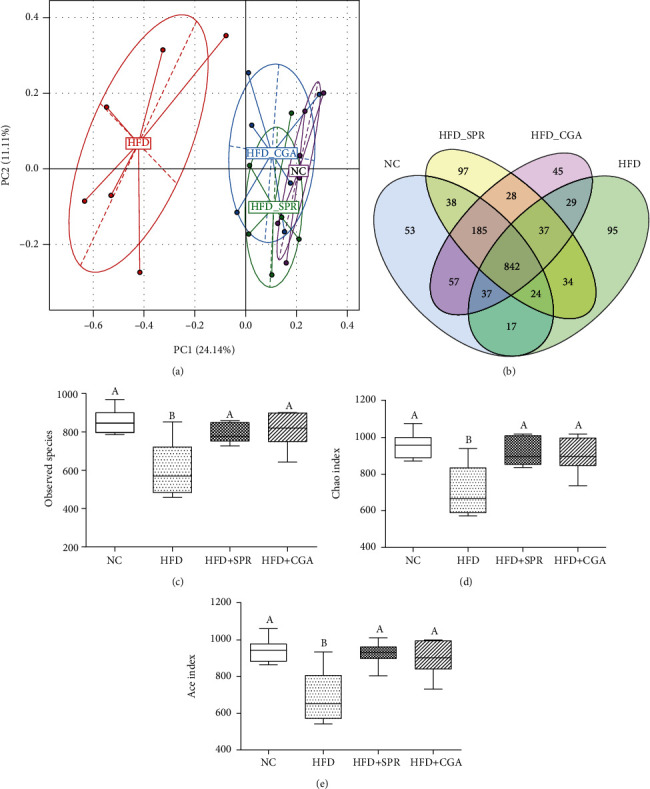
Analysis of *β*-diversity and *α*-diversity of the intestinal microflora of rats in each group. Principal component analysis (PCA) diagram (a); OTU Venn diagram (b) of rats in each group; box diagrams of Sobs (c), Chao (d), and ACE (e) of rats in each group.

**Figure 9:**
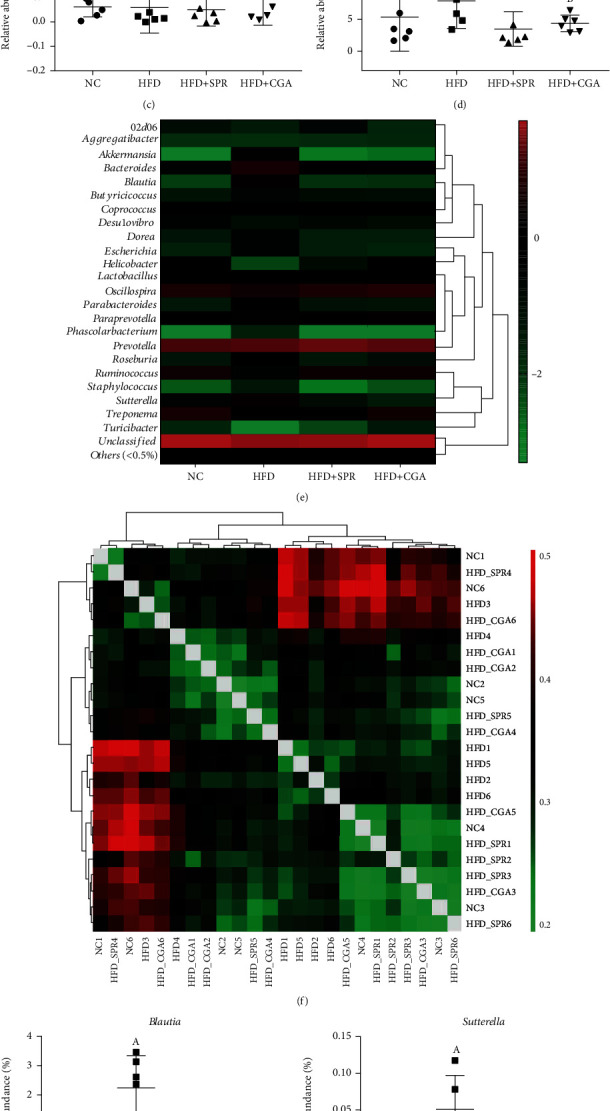
Analysis of intestinal microbial composition of rats in each group (*n* = 6). Changes in the flora composition at the level of phylum (a), changes in the horizontal heat map of genus (e), and changes in the *β*-diversity matrix heat map of genus (f). Changes in the abundance of *Tenericutes* (b), *Elusimicrobia* (c), *Proteobacteria* (d), *Blautia* (g), *Sutterella* (h), and *Akkermansia* (i) in each group.

The analysis of *β*-diversity and *α*-diversity of rat intestinal microbiota for each group is shown in Figure 8. A principal component analysis (PCA) diagram (Figure 8(a)) and a heat map of the genus-based *β*-diversity matrix (Figure 9(f)) are presented to highlight significant differences among the four groups; however, no significant differences were observed among the four groups, indicating that the experimental groups were homogeneous. Unique and overlapping OTUs, for a total of 1,618 OTUs, are represented in a Venn diagram (Figure 8(b)). Some OTUs were common among all four groups, while others were unique to a specific group. The NC, HFD, HFD+SPR, and HFD+CGA groups had 53, 95, 97, and 45 unique OTUs, respectively. As shown in the box chart (Figures 8(c)–8(e)), compared with the NC group, the Sobs, Chao index, and ACE index values of the HFD group decreased significantly, by 41.66%, 35.29%, and 37.00%, respectively. These results revealed that long-term HFD can reduce the abundance and diversity of intestinal microorganisms. Conversely, in the HFD+CGA group, the Sobs, Chao index, and ACE index values significantly increased by 34.50%, 27.89%, and 31.08%, respectively, compared with the HFD group, indicating that after CGA treatment, the richness of intestinal microorganisms returned to a level similar to that observed in the NC group.

### 3.8. Effect of Chlorogenic Acid on Intestinal Microflora

The composition of the intestinal microflora of rats in each group was analyzed and is shown in Figure 9. At the phylum level (Figure 9(a)), *Bacteroidetes*, *Firmicutes*, *Spirochaetes*, *Proteobacteria*, *Cyanobacteria*, *Verrucomicrobia*, *Tenericutes*, *Actinobacteria*, and *Elusimicrobia* were the most abundant in all four groups. Compared with the NC group, the HFD group presented a lower relative abundance of *Tenericutes* and a higher relative abundance of *Proteobacteria*. After CGA treatment, the relative abundance of *Chlorophyta* significantly increased (*P* < 0.05). Simultaneously, the abundance of *Tenericutes* increased by 65.93% and the abundance of *Elusimicrobia* decreased by 80.52%. Based on the horizontal heat map of genus (Figure 9(e)), the relative abundance of bacteria in the HFD group significantly differed from that in the NC, HFD+SPR, and HFD+CGA groups, but the relative abundance in the NC, HFD+SPR, and HFD+CGA groups tended to be consistent. Compared with the NC group, the HFD group presented a significantly higher relative abundance of *Blautia*, *Sutterella*, and *Akkermansia* (*P* < 0.05), and a lower relative abundance of *Blautia* and *Sutterella* CGA treatment.

### 3.9. Effect of Chlorogenic Acid on Short-Chain Fatty Acid Levels in Feces

**Figure 10:**
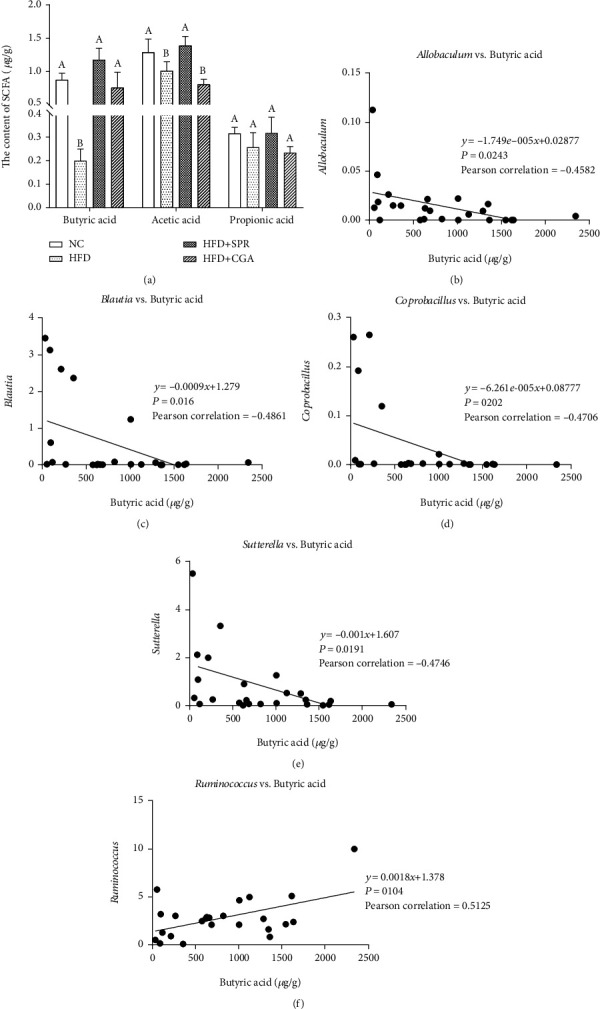
Analysis of short-chain fatty acids in the fecal of each group. (a) Butyric acid, acetic acid, and propionic acid levels in feces (*μ*g/g); correlation between butyric acid levels and the abundance of *Allobaculum* (b), *Blautia* (c), *Coprobacillus* (d), *Sutterella* (e), and *Ruminococcus* (f).

The analysis of SCFA levels in rat feces of each group is shown in Figure 10. Butyric acid and acetic acid levels were significantly lower in the HFD group than in the NC group (*P* < 0.05). Compared with the HFD group, a significant increase in butyric acid levels was observed in the CGA-treated group (*P* < 0.05). No significant difference was noted in propionic acid levels among the four groups; however, propionic acid levels were 22.57% lower in the HFD group than in the NC group. As HFD demonstrated the greatest effect on butyric acid levels, we analyzed the relative abundance of related intestinal microflora at the genus level. Furthermore, we analyzed the correlation between the abundance of intestinal microorganisms at the genus level and butyric acid levels. As shown in Figures 10(b)–10(f), butyric acid levels negatively correlated with the abundance of *Allobaculum*, *Blautia*, *Coprobacillus*, and *Sutterella* and positively correlated with the abundance of *Ruminococcus*. Therefore, we speculate that, to a certain extent, changes in the abundance and diversity of intestinal flora resulted in changes in the SCFA content.

## 4. Discussion

CGA supplementation has shown promising results for the regulation of lipid metabolism [22] and glucose homeostasis [23] via multiple mechanisms, including the promotion of lipid *β*-oxidation via PPAR-*α* [24], its antioxidant effects [25], and its capacity to improve insulin sensitivity in peripheral tissues [26]. In addition, CGA exhibits antibacterial and anti-inflammatory properties [27]. Our results indicate that a long-term HFD significantly increases body weight and induces colon inflammation in rats, compared to the NC group. Additionally, HFD-fed rats exhibited fat deposition in the liver and lipid metabolism disorders. CGA intervention improved the negative effects of HFD in rats, which is in line with the results of previous studies [12, 28, 29].

Accumulating evidence suggests that HFD has a strong effect on the gut microbiota, converting healthy gut microbiota into a dysbiotic disease-associated entity [30]. Numerous studies have shown that HFD-induced obesity leads to the development of chronic diseases, damaged intestinal mucosa, and chronic intestinal inflammation by compromising the intestinal flora of animals [30–32]. A previous study reported that approximately 30% of CGA can be absorbed into the bloodstream through the stomach and small intestine, while the remaining 70% reaches the large intestine. CGA is metabolized in the liver, and its metabolites might interact with the gut microbiota [33]. Thus, we propose two mechanisms through which CGA may exert its effects on the microbiota. One mechanism is represented by the direct entry of CGA into the large intestine, which may change the microbial structure and reduce the abundance of certain pathogenic bacterial species. For instance, the relative abundance of *Blautia* and *Sutterella* was significantly reduced (*P* < 0.05) in the intestine of CGA-treated mice compared to controls. However, our experiment found that the relative abundance of *Akkermansia* in the HFD group was significantly higher than that in the control group, which was inconsistent with the results of previous studies [34, 35]. *Akkermansia* species have been identified as mucin-degrading bacteria that reside in the mucus layer [36]. Furthermore, *Akkermansia* can help maintain the health of the digestive tract and reduce the risk of obesity, diabetes, and inflammation [37]. Interestingly, several studies have reported that resveratrol administration alters the composition and function of the gut microbiome of obese mice, and these have been characterized by a decreased abundance of *Akkermansia* [38]. Moreover, omeprazole-induced dysbiosis of the intestinal flora promotes the growth of *Akkermansia* and inhibits bifidobacterial growth, thus leading to thinning of the mucus layer through a reduced number of goblet cells in the small intestine [39]. Therefore, *Akkermansia* species may play diverse roles in the regulation of intestinal functioning, and exploring their relationship with other microorganisms in the gut environment might elucidate these roles. Additionally, we noted that, at the genus level, the abundance of *Staphylococcus* and *Escherichia* in the HFD group was higher than that in the other three groups, thereby indicating that CGA administration and the cessation of HFD could reduce the abundance of these two genera. This is consistent with previous *in vitro* CGA antibacterial test results [27, 40]. These observations describe a possible mechanism by which CGA administration may alleviate colon inflammation.

There is a second mechanism that might explain the effects of CGA. KEGG enrichment analysis revealed 11 significantly enriched signaling pathways related to amino acid and lipid metabolism in the liver, suggesting that CGA is absorbed in the stomach and small intestine and ultimately enters the liver through the bloodstream. In particular, CGA administration may significantly affect amino acid metabolism in the liver of HFD-fed obese rats, although this has not been suggested in previous studies. In our study, CGA administration downregulated the expression of phosphoserine aminotransferase (EC2.6.1.52) and D-3-phosphoglycerate dehydrogenase, which indicates lower serine biosynthesis. Conversely, the expression of L-serine/L-threonine ammonia lyase (EC:4.3.1.17,4.3.1.19) was upregulated (Fig. S1). Therefore, the synthesis of branched-chain amino acids (BCAAs) includes leucine (Leu), isoleucine (Ile), and valine (Val). Furthermore, the synthesis of glutamic acid (Glu), arginine (Arg), tyrosine (Tyr), and phenylalanine (Phe) was also affected (Fig. S2), indicating that CGA might be involved in modulating the synthesis of these BCAAs. BCAAs also act as nitrogen donors for amino acids (AAs) such as Ala, Glu, and Gln. Through signaling pathways, especially the PI3K-AKT-mTOR pathway, BCAAs are involved in the regulation of energy balance, nutrient metabolism, gut health, and immunity and thus play a key role in the etiology of diseases, such as insulin resistance or type 2 diabetes mellitus [41, 42]. In fact, metabolic imbalances in BCAAs can lead to various health problems, including diabetes and cancer [42]. Furthermore, BCAAs act as regulators, promoting intestinal development, nutrient transport, and immune-related functions, resulting in improved gut health [43–45]. Thus, a dynamic balance of BCAAs is essential for physiological and immunological health. Further, we speculate that CGA might affect bile acid excretion by modulating AA and lipid metabolism. CGA affects the composition and species abundance of the intestinal microbiota through the liver-intestine axis and alleviates HFD-induced colon mucosal injuries. Indeed, the microbial abundance of the HFD+CGA and HFD+SPR groups was similar to that of the NC group, but higher than that of the HFD group. This might be explained by the similar lipid metabolism between the HFD+CGA and HFD+SPR groups. Moreover, bile acid metabolism was also similar between the two treatment groups.

In this study, a long-term HFD led to an increase in the abundance of gram-negative *Escherichia* bacteria, which was decreased by CGA treatment. Thus, CGA treatment might be the reason for the significant decrease in serum LPS levels in the HFD+CGA group. LPS is a component of the outer wall of gram-negative bacteria, which increases intestinal tight junction permeability. Furthermore, it triggers TLR4 signal transduction, which activates the NF-*κ*B (p50/p65) pathway [46], resulting in the upregulation of inflammatory cytokines. The serum and colon levels of inflammatory cytokines observed in this study support this hypothesis. Nevertheless, our results showed that the expression level of certain proteins in the colon is not fully consistent with the expression level of the related genes in terms of mRNA accumulation. However, since mRNA molecules and proteins are the products of gene expression at different levels, this discrepancy is not surprising. In fact, efficient translation of an mRNA molecule to a protein requires editing, posttranslational processing, etc. Therefore, possibly owing to the different efficiency of transcription and translation, we did not detect perfectly corresponding mRNA and protein levels, although we measured similar trends [47]. However, we found that ELISA and western blotting yielded almost identical protein levels. At the mRNA level, the expression of the proinflammatory factor-coding genes *MCP-1* and *IL-6* in the HFD+CGA group was significantly lower than that in the HFD+SPR group. Moreover, the body weight, BMI, and Lee's index of the HFD+CGA group were significantly lower than those of the HFD+SPR group. We conclude that the partial effect of CGA administration to the HFD+CGA group led to a better outcome than that of the HFD+SPR group.

Recently, a large number of studies have shown that short-chain fatty acids (SCFAs) are effective in protecting rodents and humans from inflammation-related damage [47] and reduce the occurrence of intestinal inflammation by regulating host intestinal immunity [48]. Butyric acid, one of the fundamental SCFAs, can regulate the expression of various genes and is used as an energy source, either directly or indirectly. As an important signal transduction molecule, butyric acid can regulate gene expression by inhibiting histone deacetylases or activating G protein-coupled receptors 41 and 43 to alter the metabolic activity [49]. There are various bacterial species that produce butyric acid, including representatives of *Actinomycetes*, *Bacteroides*, *Clostridium*, *Proteus*, *Spirochetes*, and *Thermophiles* [50]. Our results reveal that the diversity of intestinal microorganisms changed after CGA treatment. The abundance of SCFA-producing bacteria increased, resulting in increased SCFA production. Compared to the NC group, the microbial diversity of the HFD+CGA group decreased, while the percentage of butyric acid-producing strains, such as *Ruminococcus*, increased significantly and showed a positive correlation with butyric acid levels. These results are in agreement with the studies discussed above.

## 5. Conclusions

To summarize, our study reveals that CGA alleviates obesity-induced colon mucosal damage. We hypothesized that chlorogenic acid changes the composition of the intestinal microbiota by regulating amino acid and lipid metabolism in SD rats or by directly acting on the microflora, thereby reducing serum LPS levels and enhancing the production of short-chain fatty acids to inhibit the development of colitis. The results provide a meaningful reference for how to adjust food composition to promote gut health.

## Figures and Tables

**Table 1:** Physical indicators of rats fed the experimental diet.

Parameter	NC	HFD	HFD+SPR	HFD+CGA
Liver (g)	14.93 ± 1.59^c^	26.80 ± 2.47^a^	18.76 ± 2.93^b^	17.83 ± 2.14^b^
Kidney (g)	3.37 ± 0.39^b^	4.04 ± 0.22^a^	3.96 ± 0.42^a^	3.29 ± 0.49^b^
Thymus (g)	0.08 ± 0.04^b^	0.15 ± 0.04^a^	0.10 ± 0.03^b^	0.12 ± 0.05^ab^
Spleen (g)	0.77 ± 0.14^b^	0.99 ± 0.14^a^	0.97 ± 0.17^a^	0.83 ± 0.09^ab^
Heart (g)	1.49 ± 0.08^c^	1.69 ± 0.13^a^	1.61 ± 0.14^ab^	1.52 ± 0.11^bc^
Lung (g)	2.19 ± 0.10^c^	3.05 ± 0.45^a^	2.74 ± 0.41^b^	2.31 ± 0.29^b^
Testicular (g)	4.30 ± 0.26^a^	3.85 ± 0.23^b^	4.37 ± 0.25^a^	4.26 ± 0.52^a^
Perirenal fat (g)	9.57 ± 2.33^c^	25.68 ± 5.32^a^	14.19 ± 2.27^b^	16.92 ± 3.14^b^
Testicular fat (g)	8.61 ± 2.53^d^	21.60 ± 3.2^a^	17.29 ± 2.36^b^	12.73 ± 4.38^c^
IgG (g/L)	1.65 ± 0.24^a^	1.26 ± 0.24^b^	1.51 ± 0.29^ab^	1.74 ± 0.26^a^
IgM (g/L)	0.12 ± 0.04^a^	0.07 ± 0.02^b^	0.10 ± 0.02^a^	0.11 ± 0.02^a^
ALT (U/L)	60.00 ± 8.72^a^	99.67 ± 20^b^	56.70 ± 7.13^a^	48.99 ± 14.48^a^
AST (U/L)	97.92 ± 14.28^a^	193.50 ± 48.17^b^	119.76 ± 33.87^a^	84.50 ± 10.24^a^
LDL-C (mmol/L)	1.39 ± 0.27^a^	1.08 ± 0.11^b^	1.37 ± 0.26^a^	1.11 ± 0.17^b^
HDL-C (mmol/L)	1.38 ± 0.28^a^	0.83 ± 0.10^b^	1.29 ± 0.26^a^	1.21 ± 0.15^a^
LDL-C/HDL-C	1.01 ± 0.10^b^	1.31 ± 0.29^a^	1.07 ± 0.17^b^	0.96 ± 0.15^b^

All data are expressed as the mean ± SD. The values in the same row with different letters are significantly different (^∗^*P* < 0.05), and the values of each group with the same letters are not significantly different (*P* > 0.05).

**Table 2:** KEGG pathway enrichment of the differential expressed genes involved in amino acid and lipid metabolism between the HFD and HFD+CGA groups.

Serial	Pathway	DEGs	*P* value	*Q* value	Pathway ID
4	Glycine, serine, and threonine metabolism	6/42	0.00004	0.00228	ko00260
6	Steroid biosynthesis	4/26	0.00065	0.02269	ko00100
7	Cysteine and methionine metabolism	5/58	0.00205	0.06087	ko00270
8	Valine, leucine, and isoleucine biosynthesis	2/5	0.00234	0.06087	ko00290
9	Linoleic acid metabolism	4/41	0.00370	0.08540	ko00591
11	Glycerophospholipid metabolism	6/106	0.00626	0.11017	ko00564
12	Phenylalanine, tyrosine, and tryptophan biosynthesis	2/8	0.00636	0.11017	ko00400
18	Arachidonic acid metabolism	6/119	0.01079	0.12472	ko00590
25	Alanine, aspartate, and glutamate metabolism	3/41	0.02574	0.21416	ko00250
30	Arginine biosynthesis	2/23	0.04930	0.33080	ko00220
31	Phenylalanine metabolism	2/23	0.04930	0.33080	ko00360
37	Bile secretion	4/95	0.06103	0.34306	ko04976

KEGG pathway enrichment of the differential expressed genes involved in amino acid and lipid metabolism between the HFD and HFD+CGA groups. Notice: we just showed the *P* value is equal or smaller than 0.05 about the pathway of amino acid and lipid metabolism, and bile secretion.

## Data Availability

The datasets for this research can be found in NCBI SRA, https://www.ncbi.nlm.nih.gov/Traces/study/?acc=PRJNA658840.

## Supplementary Materials

Supplementary Materials Supplementary Description: Figure S1: KEGG pathway enrichment of the differential expressed genes of biosynthesis of amino acids. Upregulated steps are marked with red lines and downregulated steps with green lines. Figure S2: KEGG pathway enrichment of the differential expressed genes of glycine, serine, and threonine metabolism.
