# Supplementary figures and images for: Chlorogenic Acid Alleviates Colon Mucosal Damage Induced by a High-Fat Diet via Gut Microflora Adjustment to Increase Short-Chain Fatty Acid Accumulation in Rats
Source: Oxid Med Cell Longev. 2021 Feb 1;2021:3456542. doi: 10.1155/2021/3456542 (PMC7889347; doi:10.1155/2021/3456542)

## BIOSYNTHESIS OF AMINO ACIDS

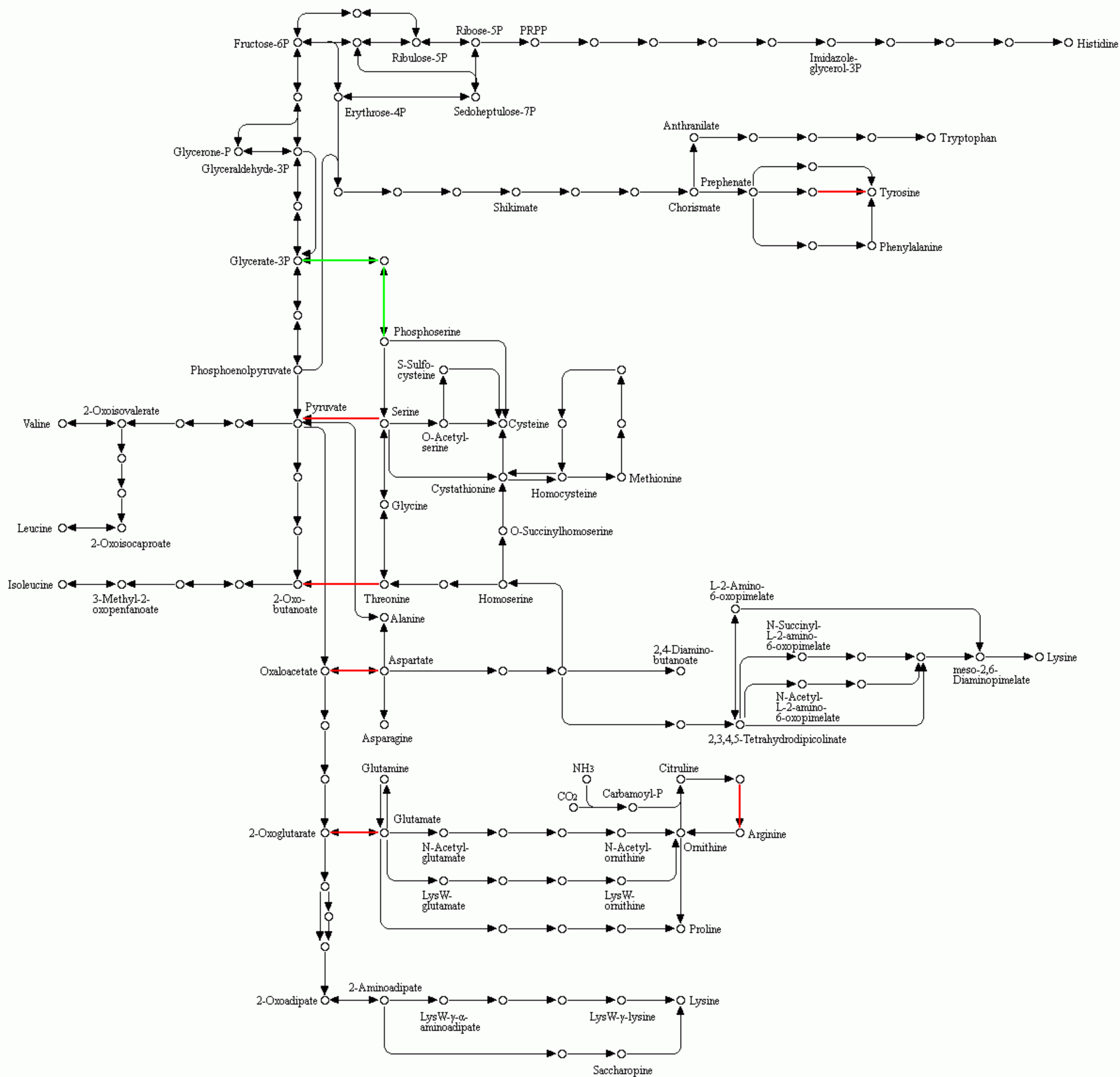

Supplement: Supplementary Materials — Supplementary Description: Figure S1: KEGG pathway enrichment of the differential expressed genes of biosynthesis of amino acids. Upregulated steps are marked with red lines and downregulated steps with green lines. Figure S2: KEGG pathway enrichment of the differential expressed genes of glycine, serine, and threonine metabolism. [file 3456542.f1.zip › 3456542.f1/1 KEGG pathway enrichment of the differential expressed genes of Biosynthesis of Amino acids. Up-regulated steps are marked with red lines and down-regulated steps with green lines..pdf]

# GLYCINE, SERINE AND THREONINE METABOLISM

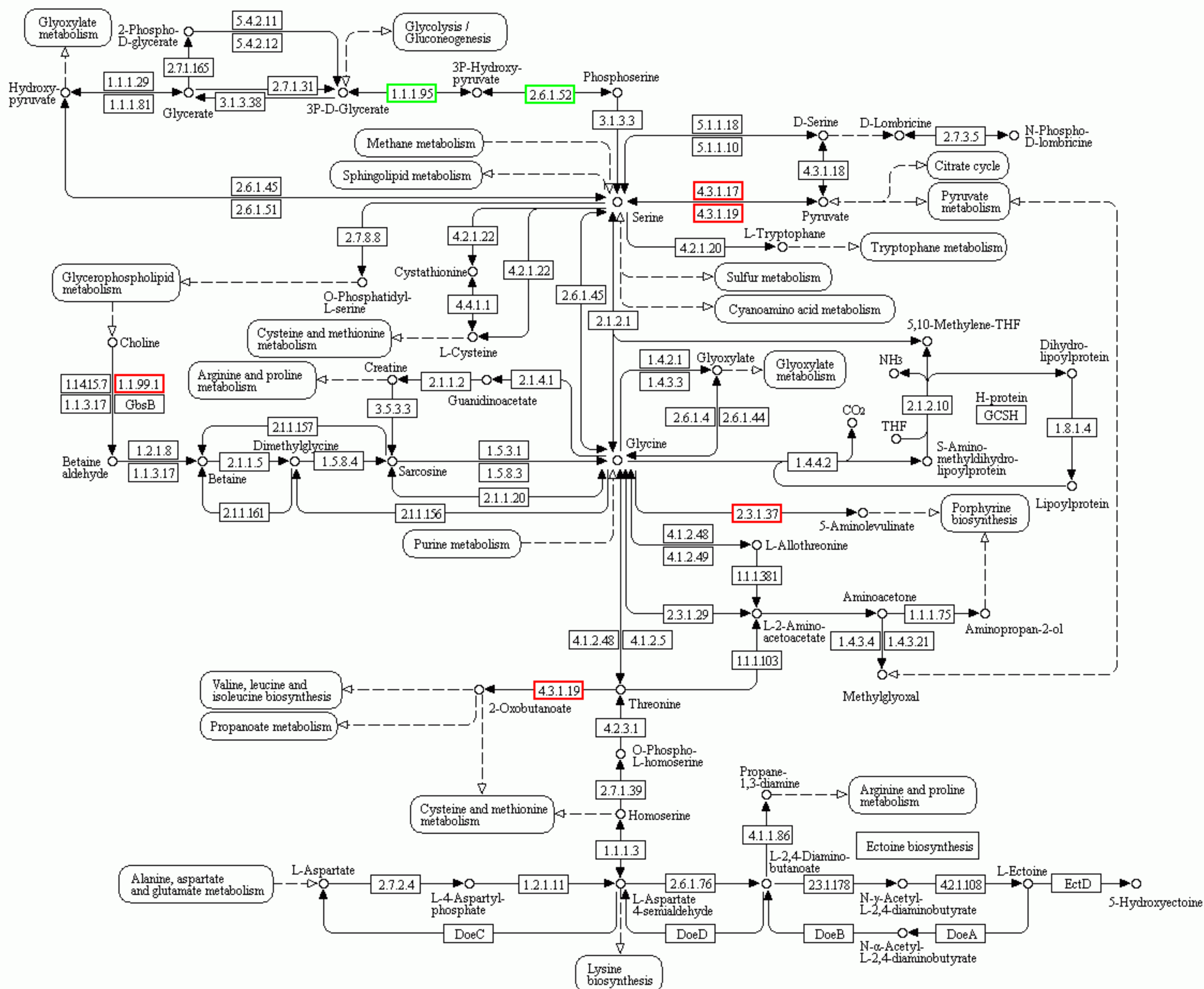

Supplement: Supplementary Materials — Supplementary Description: Figure S1: KEGG pathway enrichment of the differential expressed genes of biosynthesis of amino acids. Upregulated steps are marked with red lines and downregulated steps with green lines. Figure S2: KEGG pathway enrichment of the differential expressed genes of glycine, serine, and threonine metabolism. [file 3456542.f1.zip › 3456542.f1/2 KEGG pathway enrichment of the differential expressed genes of Glycine, Serine and Threonine metabolism.pdf]
